# BEHAVIORAL CHARACTERISTICS OF CHILDREN WITH SICKLE CELL DISEASE

**DOI:** 10.1590/1984-0462/2021/39/2019341

**Published:** 2020-08-05

**Authors:** Fellipe Bondança Pereira, Glaura César Pedroso, Rosa Miranda Resegue, Marcos Vinicius Vieira Ribeiro, Mary Hokazono, Josefina Aparecida Pellegrini Braga

**Affiliations:** a Universidade Federal de São Paulo, São Paulo, SP, Brazil.

**Keywords:** Sickle cell disease, Child behavior, Socioeconomic factors, Doença falciforme, Comportamento infantil, Fatores socioeconômicos

## Abstract

**Objective:**

To evaluate sociodemographic and clinical aspects of children with sickle cell disease (SCD) and their behavioral characteristics.

**Methods:**

Interview with parents of patients with SCD from four to ten years old, addressing socioeconomic aspects and other health conditions, and using the Strengths and Difficulties Questionnaire (SDQ). Clinical data were obtained from medical records. Exclusion criteria were the use of hydroxyurea, previous diagnosis of stroke, chronic encephalopathy and/or intellectual disability.

**Results:**

45 patients (19 girls and 26 boys) were assessed. The median age was seven years. Diagnosis of SCD: 26 hemoglobinopathy SC; 19 hemoglobinopathy SS. Socioeconomic class: D: 24.4%; C2: 44.4%; C1: 28.9%; B2: 2.2%. Clinical history: acute chest syndrome: 40%; transfusions: 66.7%; hospitalizations: 82.2%. SDQ findings: 88.9% clinical impact (emotional subscale: 68.9%); total score: impact in 48.9%. It was not possible to establish a relation between the severity of the disease and the results of the SDQ. Regarding socioeconomic class: among individuals of classes B2 and C1, 21.4% had impact at the total score; in classes C2 and D, this percentage was 61.3%. Regarding the schooling of the head of the family, with Elementary School at least, 39.3% of the children had impacts; for fewer education, this percentage was 64.7%.

**Conclusions:**

Behavioral impacts are highly prevalent in children with SCD. Individuals in socioeconomic classes C2 and D suffered more behavioral impacts than individuals in classes B2 and C1.

## RESUMO

**Objetivo:**

Avaliar aspectos sociodemográficos e clínicos de crianças com doença falciforme (DF) e suas características comportamentais.

**Métodos:**

Aplicação de entrevista sobre aspectos socioeconômicos e outras condições de saúde e do questionário de capacidades e dificuldades (SDQ) em pais de pacientes de quatro a dez anos com DF, em um ambulatório de referência. Dados clínicos foram obtidos dos prontuários médicos. Critérios de exclusão: uso de hidroxiureia, diagnóstico prévio de acidente vascular cerebral, encefalopatia crônica e/ou deficiência intelectual.

**Resultados:**

Analisados 45 pacientes (19 meninas e 26 meninos). Mediana de idade=7 anos. Diagnóstico da DF=26 hemoglobinopatia SC; 19 hemoglobinopatia SS. Classe econômica (SES): D=24,4%; C2=44,4%; C1=28,8%; B2=2,2%. Antecedentes clínicos: síndrome torácica aguda=40%; transfusões=66,7%; internações=82,2%. Achados SDQ=88,9% alteração clínica (subescala emocional=68,9%); pontuação total=alterada em 48,9%. Não foi possível estabelecer relação entre gravidade da doença e os resultados do SDQ. Com relação à SES, entre indivíduos das classes B2 e C1, 21,4% tiveram alteração na pontuação total; nas classes C2 e D, esse percentual foi de 61,3%. Quanto à escolaridade do chefe da família, com no mínimo ensino fundamental completo, 39,3% das crianças tiveram alteração; para menor escolaridade, esse percentual foi 64,7%.

**Conclusões:**

Alterações comportamentais são altamente prevalentes em crianças com DF. Indivíduos das classes C2 e D tiveram mais alterações comportamentais em relação aos indivíduos das classes B2 e C1.

## INTRODUCTION

Sickle cell disease (SCD) is one of the most frequent genetic conditions in Brazil. It is a monogenic disease resulting from a mutation in the gene responsible for the production of the hemoglobin A beta globin, which determines changes in the resulting hemoglobin molecule (hemoglobin S).^1^ In children, SCD is the main cause of cerebrovascular accidents (CVA), occurring in 8 to 17% of patients.^2^ We must also highlight the possibility of the occurrence of silent cerebral infarctions, which affects approximately 25% of children with sickle cell disease until the age of six, and up to a third of these children until the age of 14, which can lead to worse performance in cognitive tests when compared to the general population, specific functional deficiencies and impaired school performance.^3^ Additionally, vaso-occlusive crises are the main clinical symptom of SCD and the first cause of hospitalization by patients, so that about 60% of patients with SCD have at least one episode of severe pain per year related to vaso-occlusive crises, and the first episodes can occur at around six months of age.^2^ ^,^ ^3^

Considering these possible clinical manifestations in pediatric patients, studies demonstrate the relation of SCD with behavioral, emotional, academic and social interaction problems in children, which can be attributed not only to issues pertinent to the chronicity of the disease (such as missing classes due to prolonged periods of hospitalization and the need for special care), but also to specific issues of SCD (such as the suffering related to unpredictable painful crises and the cognitive deficit resulting from silent cerebral infarctions, for example).^2^ ^,^ ^4^

The analysis of child development and behavior must consider the existing interactions between the child’s individual characteristics and the environmental contexts to which they are exposed. In this sense, the demands that SCD imposes on the family as from at the diagnosis lead to changes in the care of the child and changes in the family routine, which can lead to situations of overprotection and exacerbation of the dependence of the child with SCD in relation to their caregivers.^5^ ^,^ ^6^

There are several instruments for assessing emotional and behavioral problems in children and adolescents. Among them, there is the strengths and difficulties questionnaire (SDQ), proposed by Goodman in 1997, designed to be a short, simple and clinically useful instrument.^7^ ^,^ ^8^ ^,^ ^9^ Thus, the objectives of the present study were to evaluate , according to the SDQ, the behavioral characteristics of children with SCD followed up in a pediatric hematology referral clinic and to analyze possible influences of sociodemographic, clinical and laboratory characteristics on their behavior.

## METHOD

This is a cross-sectional study, with convenience sampling, in which the SDQ was applied to the parents of patients from four to ten years old who were diagnosed with SCD and attended the referral clinic during the study period (November 2018 to March 2019). Additionally, some questions related to other issues relevant to the study were asked, such as the socioeconomic conditions of the family, other health conditions of the child/adolescent and their school performance.

Due to the socio-cultural aspects and differences that exist in our environment, which could lead to differences or difficulties in understanding the questions, the questionnaire was applied in an interview with the parents.

Clinical and laboratory data were obtained from the children’s medical records, which were used to characterize the severity of the disease, in order to assess the impact of the severity of SCD on children’s behavior. The occurrence of events of acute chest syndrome, blood transfusions and hospitalizations; the current hemoglobin; current white blood count; and the current dosage of lactic dehydrogenase (LDH) were collected.

The following exclusion criteria were adopted: patients with a previous diagnosis of stroke, chronic encephalopathy and intellectual disability; and patients using hydroxyurea. The latter were excluded because of the drug’s impact on quality of life and the number of crises resulting from SCD. Thus, the inclusion of these patients could create very divergent groups, which could not be comparable.

Parents or guardians of children who had changes identified in the SDQ and in the SDQ impact supplement were personally notified in the return visits of children to the outpatient clinic, being referred for evaluation in the Psychiatric department of the referral unit.

The study started after approving the project by the Research Ethics Committee of Universidade Federal de São Paulo (UNIFESP-CEP nº 1.073/2018). All parents (or guardians) and children participating in the study received information and clarifications regarding its objectives and possible risks, and only those who signed the informed consent form (ICF) before the study began and, in the case of children over five, the term of consent (TC) participated on research.

The instrument used was the SDQ, which is free to use. Developed in 1997 by Robert Goodman, of the London Institute of Psychiatry, it is a short, simple and clinically useful tool for tracking mental health problems and assessing the behavior of children and adolescents. The questionnaire has a total of 25 items, divided into five scales with five items each, which aim to cover issues pertinent to emotional symptoms, conduct problems, hyperactivity, relationship problems with colleagues and prosocial behavior. For each item, the person responsible for filling in indicates whether it is “false”, “somewhat true” or “true”, with a score from zero to two, depending on the answer. The results are interpreted by evaluating the scores of all scales together and of each separately, classifying the results as “normal”, “borderline” and “abnormal” (after the scores for these classifications were determined 80% of the children in the community were in compliance with the values considered “normal”, 10% “borderline” and 10% “abnormal”).^7^ ^,^ ^8^ ^,^ ^9^ ^,^ ^10^

There are different versions of the SDQ according to the patient’s age group and to whom is answering the questionnaire (parents or educators, teachers or the patient themselves). For the present study, the version for parents of patients from four to 16 years of age was used.

Additionally, the SDQ impact supplement was used to complement the study results. This supplement adds eight questions to the questionnaire, allowing assessment of the chronicity of the problems and their perceived impacts on the daily activities of the child or adolescent. It is worth mentioning that the SDQ is one of the most widely used research tools for screening problems related to the mental health of children and adolescents, it is available for free (http://www.sdqinfo.com) and has already been translated and validated in more than 40 countries.^11^ In Brazil, it was translated and validated for a population study carried out in the Southeastern Region and used in several other studies referring to the mental health problems of children and adolescents.^8^ ^,^ ^9^ ^,^ ^12^ ^,^ ^13^ ^,^ ^14^ ^,^ ^15^

Complementarily to the SDQ, parents were asked questions related to the family’s socioeconomic conditions, other comorbidities of the child and the child’s school performance. To assess the family’s socioeconomic conditions, the parameters considered in the Brazil Economic Classification Criterion (Brazil Criterion), from the Brazilian Association of Research Companies (*Associação Brasileira de Empresas de Pesquisa* - ABEP) were used as a reference. The Brazil Criterion by ABEP came into effect in 2015 and was developed by Professors Wagner Kamakura (Rice University) and José Afonso Mazzon (Faculty of Economics, Administration and Accounting at Universidade de São Paulo) based on the Family Budget Survey by the Brazilian Institute for Geography and Statistics (*Instituto Brasileiro de Geografia e Estatística* - IBGE). By means of parameters that take into account the number of comfort items (such as cars, appliances, bathrooms) in the family home, access to running water, street paving and the education of the head of the family, the Brazil Criterion by ABEP estimates the socioeconomic strata to which the family belongs.^16^ Thus, these parameters in the present study allow the assessment of the possible influence of the socioeconomic conditions of the families of patients with SCD on their behavioral characteristics.

The data were evaluated using the online software available at: <https://www.socscistatistics.com/tests/what_stats_test_wizard.aspx>. The χ^2^ test was used as statistical methods to assess categorical variables and Student’s t-test to compare means between groups. For both tests, a significance level of 5% (p <0.05) was adopted.

## RESULTS

Over the period of data collection, 45 parents of patients between four and ten years old (19 girls and 26 boys) were interviewed, with a median age of seven years. Patients with SCD hemoglobinopathy represented 57.8% (26) of the total, whereas patients with SS hemoglobinopathy, 42.2% (19).

As for the questionnaire of Brazil Criterion by ABEP, it was possible to observe the predominance of classes C and D among patients (class D=24.4%; C2=44.4%; C1=28.9%; B2=2.2%).

According to the SDQ criteria, 88.9% of the children were identified as having impact in some of the parameters evaluated by the questionnaire, and another 6.7% were classified as “borderline”, so that only two children were defined as “without impact”. These numbers diverged significantly from those found when assessing the parents’ responses to the impact supplement, which showed the percentage of 20% of patients with some impact. Since the impact supplement mainly evaluates the parents’ perception of their child’s behavior, this discrepancy may demonstrate their lack of perception of the problem. Table 1 contains this data in detail.

**Table 1:** São Paulo, 2018. Distribution of patients according to the results of the capacities and difficulties questionnaire (SDQ). city of São Paulo, 2018.

Parameter	No impact		Borderline		With impact	
	n	%	n	%	n	%
Emotional problems	9	20.0	5	11.1	31	68.9
Conduct problems	16	35.6	5	11.1	24	53.3
Hyperactivity	22	48.9	8	17.8	15	33.3
Peer problems	23	51.1	12	26.7	10	22.2
Prosocial behavior	45	100	0	0	0	0
Total score	10	22.2	13	28.9	22	48.9
Impact supplement	34	75.6	2	4.4	9	20.0
Any impact	2	4.4	3	6.7	40	88.9

Among the specific scales evaluated in the SDQ, the emotional symptoms and the conduct problems showed a higher percentage of patients with impact, with 68.9 and 53.3%, respectively.

Of the 45 children studied, 40% had a history of acute chest syndrome, 66.7% had a history of transfusions and 82.2% were hospitalized. With such data, these parameters were used as criteria for disease severity, to ascertain any differences between the behavioral characteristics of children according to severity. Table 2 shows the distribution of patients according to the history of occurrence of these parameters and their results in the SDQ, and Table 3 compares the total questionnaire score of the groups with and without these histories. As it can be seen in these tables, there was no statistically significant association between the severity of the disease and the impact perceived in the SDQ.

**Table 2:** Distribution of patients according to severity parameters and results of the strengths and difficulties questionnaire (SDQ). City of São Paulo, 2018.

Parameter	Hospitalization history		Transfusions history		ACS history	
	n with impact	%	n with impact	%	n with impact	%
Emotional problems	25	67.7	19	63.3	13	72.2
Conduct problems	22	59.5	18	60.0	10	55.6
Hyperactivity	14	37.8	10	33.3	4	22.2
Peer problems	8	21.6	7	23.3	4	22.2
Prosocial behavior	0	0	0	0	0	0
Total score	19	51.4	15	50.0	8	44.4
Impact supplement	8	21.6	5	16.7	2	11.1
Any impact	32	86.5	26	86.7	16	88.9

ACS: acute chest syndrome.

**Table 3:** São Paulo, 2018. Averages of the total score of the strengths and difficulties questionnaire (SDQ) according to severity parameters. City of São Paulo, 2018.

Severity parameter	Average of total SDQ score*^,^**	Patients with impact*** (%)
Hospitalization history		
Yes	17.5	51.4
No	15.6	37.5
p	0.439	0.477
Transfusions history		
Yes	17.2	50.0
No	17.0	46.7
p	0.905	0.833
ACS history		
Yes	17.0	44.4
No	17.3	51.9
p	0.891	0.626

*SDQ classification: 0 to 13=“normal”; 14 to 16=“borderline”; 17 to 40=“abnormal”; **p calculated by Student’s t-test; ***p calculated by χ^2^ test; ACS: acute chest syndrome.

School problems of the children studied were assessed with an open question that asked whether the parents had already received complaints from the school regarding their child (and, if the answer was affirmative, what type of complaint). Of the total of interviews carried out, 24 parents reported having already received complaints from the school (53.3% of the total), and 15 of the corresponding children had impact in the parameter “total score” of the SDQ (68.2% of the total with school problems), and only three were classified as without impact according to this parameter (and six as “borderline”). Most complaints were related to behavioral problems (17 cases), followed by attention problems (seven cases). Learning disability was reported as a problem for only one child.

The analysis of the association between socioeconomic class and parents’ education with SDQ performance is described in Table 4. There was a statistically significant difference between groups, with a higher percentage (61.3%) of patients with impact in classes C2 and D, when compared to classes C1 and B2 (21.4%).

**Table 4:** Results of the strengths and difficulties questionnaire - SDQ) according to socioeconomic classes. City of São Paulo, 2018.

Socioeconomic class	Average of total SDQ score*^,^**	Patients with impact*** (%)
C1 and B2	14.5	21.4
C2 and D	18.4	61.3
p	0.048	0.013

*SDQ classification: 0 to 13=“normal”; 14 to 16=“borderline”; 17 to 40=“abnormal”; **p calculated by Student’s t-test; ***p calculated by χ^2^ test.

This trend was repeated when the patients’ results were compared according to the education of the head of the family, who, according to the criteria of ABEP, is the person who contributes with most of the household income. In the group with complete elementary school, 39.3% showed some impact (average of the total SDQ score=16.8), whereas in the group without complete elementary school the percentage was 64.7% (average of the total SDQ score=17.8). However, this difference was not statistically significant, which may be related to the small sample size.

## DISCUSSION

Child development and behavior are influenced by interactions between the child’s individual characteristics and the environmental contexts to which they are exposed. Dias et al.^2^ and Lorencini and De Paula^4^ studied children with SCD and indicated the apparent impact of the disease on the behavior and development of these children. More broadly, Hysing et al.^17^ surveyed more than seven thousand children with various chronic diseases and demonstrated the impacts on their behaviors with the SDQ.

In agreement with these observations, the results of the present study pointed to a great impact of SCD on the behavior and development of patients with the disease, seen that 88.9% of the children included presented impact in some of the parameters evaluated by the questionnaire. Taking the care of pondering the large differences between the populations of the two studies, it is possible to compare these results with those obtained by Hysing et al.^17^. In the present study, a much higher percentage of children with behavioral impact (total SDQ score criterion=48.9 vs. 19.4%) was observed. This difference is probably related to specific characteristics of SCD (such as the unpredictability of the disease, painful crises and restrictions imposed by the disease) and to other aspects of the life of the different populations of the studies (such as socioeconomic status, emotional support and quality of life).

Additionally, when comparing the results of the present study with those obtained by Stivanin et al.,^18^ who used the SDQ to assess behavioral problems in 74 healthy children in the city of São Paulo, a much higher percentage of children with impact in some of the parameters evaluated by the SDQ in the present study (88.9 vs. 33.8%) was found. As in the comparison with the study by Hysing et al.^17^, these differences must be pondered, since the groups are quite different. Nonetheless, these analyses point to a strong impact of SCD on the behavior of the affected children. Similarly, although detailed information about school problems was not the focus of this study, the data obtained suggest that the most relevant factor for these problems possibly is the behavioral issues of these children.

Despite these observations, it was not possible to establish a statistically significant relation between the severity of SCD and the behavioral impacts of children. Perhaps, this impossibility is related to the sample size used in the study, which was limited, and that the sole fact that children have the disease represents demands for them and their family, thus culminating in impacts on behavior, even in the less serious forms of SCD, so that other aspects of these people’s lives have an even greater influence than the severity of the disease. It is worth mentioning, in this sense, that the demands that SCD imposes on the family as from the diagnosis lead to changes in the child’s care and in the family routine, which can lead to situations of overprotection and exacerbation of the child’s dependence on their caregiver.

In line with the investigation of aspects that may be involved in child behavior and development, Rodriguez et al.^19^ studied 805 children in the municipality of São Luiz, state of Maranhão, pointing out the influence of socioeconomic and demographic conditions on mental health problems. These results point in the same direction as those from the present study, which demonstrated statistically significant differences between the total SDQ score of children from different socioeconomic classes. Thus, it can be inferred that SCD seems to be an important factor for behavioral changes in children, but the socioeconomic conditions of the family may have a more important impact on the behavior and development of these children than the severity of the disease itself.

The present study had limitations due to the sample size and because it is a cross-sectional study, which does not allow assessing associations between variables over time, as well as the absence of a control group of healthy children with equal socioeconomic characteristics. The findings point to the need for further studies with larger samples, different populations of children and designs that make it possible to assess the frequency of impacts at different times throughout life and the evolution of the disease. The knowledge of these impacts is important for the development of strategies for the qualification of assistance to children with SCD, especially for those with a more important social vulnerability.
